# High-Resolution Imaging of Photocatalytic Activity and Charge Separation by Multimodal Photo-SECM

**DOI:** 10.1021/acs.accounts.6c00429

**Published:** 2026-09-02

**Authors:** Ziyuan Wang, Shu Hu, Michael V. Mirkin

**Affiliations:** † Department of Chemistry and Biochemistry, 14781 Queens College-CUNY, Flushing, New York 11367, United States; ‡ Department of Chemical and Environmental Engineering, 5755 Yale University, New Haven, Connecticut 06510, United States; § Advanced Science Research Center, CUNY, New York, New York 10031, United States

## Abstract

Photocatalytic processes are central to many energy and environmental applications. Understanding the fundamental parameters that govern these reactions at the nanoscale is essential for the rational design of solar energy conversion systems and sustainable chemical synthesis. In nanostructured photocatalysts, the charge-transfer rate and direction and catalytic activity can vary within nanometer-scale distances across different sites—even within a single nanoparticle—due to structural, compositional, and electronic inhomogeneities. Traditional techniques provide bulk-averaged data, thereby obscuring such intrinsic spatial variability and limiting mechanistic insight. Photo-scanning electrochemical microscopy (photo-SECM) has proven useful for mapping local catalytic activity through current-based measurements, but it lacks the capacity to directly probe surface potentials and the driving force for light-induced charge-transfer and light-driven catalytic processes. The high complexity of underlying mechanisms, which include photogeneration, separation, and transport of charge carriers, as well as interfacial charge-transfer processes, necessitates multimodal correlative experimental characterizations. However, widely employed characterization techniques such as surface photovoltage spectroscopy (SPV) and photoemission electron microscopy (PEEM) operate under vacuum or in air, failing to capture the steady-state driving forces for light-induced redox reactions.

A recently developed nanoscale photo-SECM methodology has enabled simultaneous interrogation of interfacial electron-transfer reactions and charge separation in particulate and two-dimensional photo­(electro)­catalysts. In this Account, we discuss the development of a multimodal SECM technique that combines surface potentiometry with amperometric reaction rates mapping to directly resolve the steady-state energetic landscape, along with the spatial activity distribution within individual catalytic domains. This correlative approach enables simultaneous access to both kinetics (reaction flux) and thermodynamics (local potential), providing a more complete description of photocatalytic function. The surveyed applications include probing charge separation in a monolayer in-plane heterojunction, high-resolution activity mapping of heterogeneous photocatalysts by the tunnelling mode of photo-SECM, and amperometric/potentiometric photo-SECM measurements of photoelectrochemical processes in nanostructured photocatalysts *in operando*. The ability of multimodal SECM to resolve steady-state interfacial energetics and kinetics under catalytic turnover was essential for probing a novel planar photocatalyst in which flux-responsive charging of cocatalyst surface dynamically sustains the high local driving force while maintaining efficient separation, accumulation, and utilization of photogenerated electrons and holes.

Future developments may further extend this platform toward time-resolved and dynamic measurements of charge separation and interfacial processes, as well as perturbing the system with dynamically applied bias and illumination conditions, thus offering additional insight into transient photophysical and photochemical phenomena on the μs–s time scale and bridging the gap between the short time scale of ultrafast spectroscopies (fs–ps) and steady-state photo-SECM kinetic measurements. We envision this technique to enable nanoscale interrogation of surface potentials, reaction rates, band energetics, and driving forces, thereby guiding the design of photocatalysts and active sites with tailored geometries and compositions to access a wide range of redox chemistries. Along with these localized probing techniques, integrating machine-learning algorithms for autonomous probe control, approach-curve analysis, and real-time data-driven tip positioning may further transform potential-sensing photo-SECM into a self-driving platform for highly reproducible, autonomous interrogation of photocatalytic interfaces.

## Graphical abstract

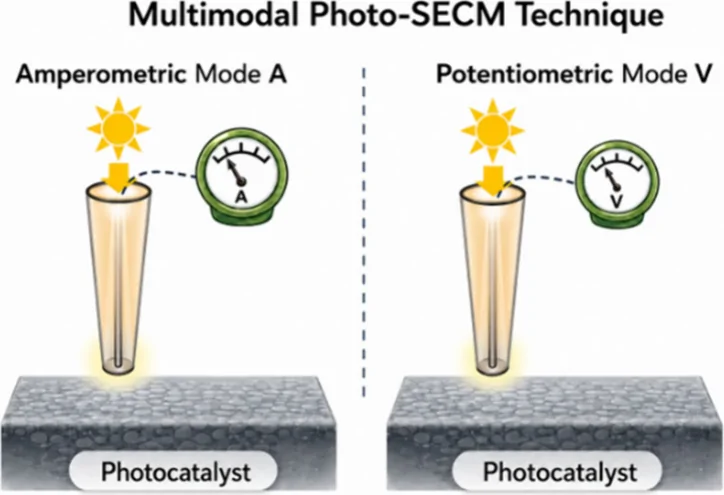

## Key References

-

Huang, Q. ; Wang, Z. ; Liu, R. ; Yao, H. ; Ni, C. ; Bo, T. ; Wu, S. ; Sun, F. ; Fan, F. ; Mirkin, M. V. In Situ Imaging Reveals Efficient Charge Separation in Monolayer MoS_2_–WS_2_ Type-II Heterojunctions. J. Am. Chem. Soc. 2026, 148, 8417–8422 10.1021/jacs.5c19244PMC1296452441719087.^1^ The combination of photo-SECM with surface photovoltage microscopy (SPVM) and photoluminescence (PL) spectroscopy provided spatially resolved, direct visualization of charge separation in 2D type-II heterojunctions and offered critical guidelines for the rational design of high-performance lateral TMDs photocatalysts and optoelectronic devices.
-

Bo, T. ; Su, H. ; Wang, Z. ; Bae, J. H. ; Askarova, G. ; Hu, S. ; Mirkin, M. V. Probing Charge-Transfer Processes in Pt/TiO_2_ Photocatalyst by Amperometric/Potentiometric Photo-SECM. Proc. Natl. Acad. Sci. U.S.A. 2026, 123, e2527861123 10.1073/pnas.2527861123PMC1303789541886380.^2^ This work demonstrated that amperometric/potentiometric photo-SECM can sequentially measure product fluxes and surface potentials at the same location with ∼10 nm resolution under *operando* conditions.
-

Ma, X. ; Li, Z. ; Amirifard, M. ; Ennis, Q. ; Su, H. ; Shang, J. ; Zhang, W. ; Yanagi, R. ; Lee, Y. ; Kortshagen, U. ; Hu, S. Enhanced Photocatalytic Charge Separation in Coating-Protected Porous Silicon Quantum Dot Films With Stability Approaching 100 h. Adv. Funct. Mater. 2026, 36, e02774.^3^ In this work, charge separation is unequally shown through purely different kinetics of electron transfer vs hole transfer, as compared to all reported literature, where the local band energetics are different and all vary locally, however small they are. This observation motivates the need for quantitative probing of local kinetics and energetics by potentiometric photo-SECM as discussed in this manuscript.
-

Bo, T. ; Ghoshal, D. ; Wilder, L. M. ; Miller, E. M. ; Mirkin, M. V. High-Resolution Mapping of Photocatalytic Activity by Diffusion-Based and Tunneling Modes of Photo-Scanning Electrochemical Microscopy. ACS Nano 2025, 19, 3490–3499.10.1021/acsnano.4c13276PMC1178103139792635 ^4^ In this work, the tunneling mode of photo-SECM was introduced and employed for ultrahigh-resolution mapping of surface reactivity and the differences in the lateral charge transfer rate in two-dimensional photocatalysts.

## Introduction

Photocatalysis harnesses solar energy to drive key redox transformations, including water splitting,^5^ CO_2_ reduction,^6^ and pollutant degradation.^7^ Understanding the driving forces that govern photocatalytic reactions remains a central challenge in solar energy conversion and chemical synthesis.^8–10^ The efficiency of these processes depends not only on the generation of photocarriers, but also on their separation, transport, recombination, and eventual transfer across semiconductor–electrolyte interfaces.^11–14^ In practice, these processes are rarely spatially uniform. Even within a single particle, different facets, cocatalyst domains, heterojunction interfaces, and defect-rich regions can exhibit distinct carrier populations, charge-transfer kinetics, and local energetic landscapes.^15–20^ Yet our current understanding of photocatalytic materials still relies largely on bulk measurements, which average the local variations and mask important spatial differences within and across particles, thereby providing only limited insight into the local structure–function relationships that govern photocatalytic performance.

SECM with micrometer-scale probes has been widely used to investigate photoelectrochemical processes and to screen photocatalytic materials.^21–27^ However, the spatial resolution of micrometer-scale probes is not high enough to distinguish the behavior of individual particles or different reactive regions within a particle. Recent advances in through-tip illumination have enabled photo-SECM to be extended to the nanoscale, where the probe can function simultaneously as a light guide and an electrochemical sensor.^28^ Because illumination is confined to the region of electrochemical detection, this configuration avoids system-wide concentration changes induced by global illumination and helps preserve local spatial resolution, enabling photocatalytic processes to be studied at the level of individual particles, facets, and heterointerfaces.^1,2,4,29–32^

Feedback and substrate generation–tip collection (SG–TC) modes of photo-SECM have previously been used for studying photocatalysts and photoelectrocatalysts.^15,33–35^ In the feedback mode (Figure 1A), the tip current (*i*_T_) reflects the ability of the substrate surface to regenerate a redox mediator produced at the tip; the efficient mediator recycling gives rise to positive feedback (i.e., *i*_T_ increases with decreasing separation distance, *d*), whereas diffusional blocking by the substrate produces negative feedback (i.e., *i*_T_ decreases as the tip approaches the sample), making this mode particularly useful for locating individual particles and topography imaging (Figure 1F).^30^

**Figure 1:**
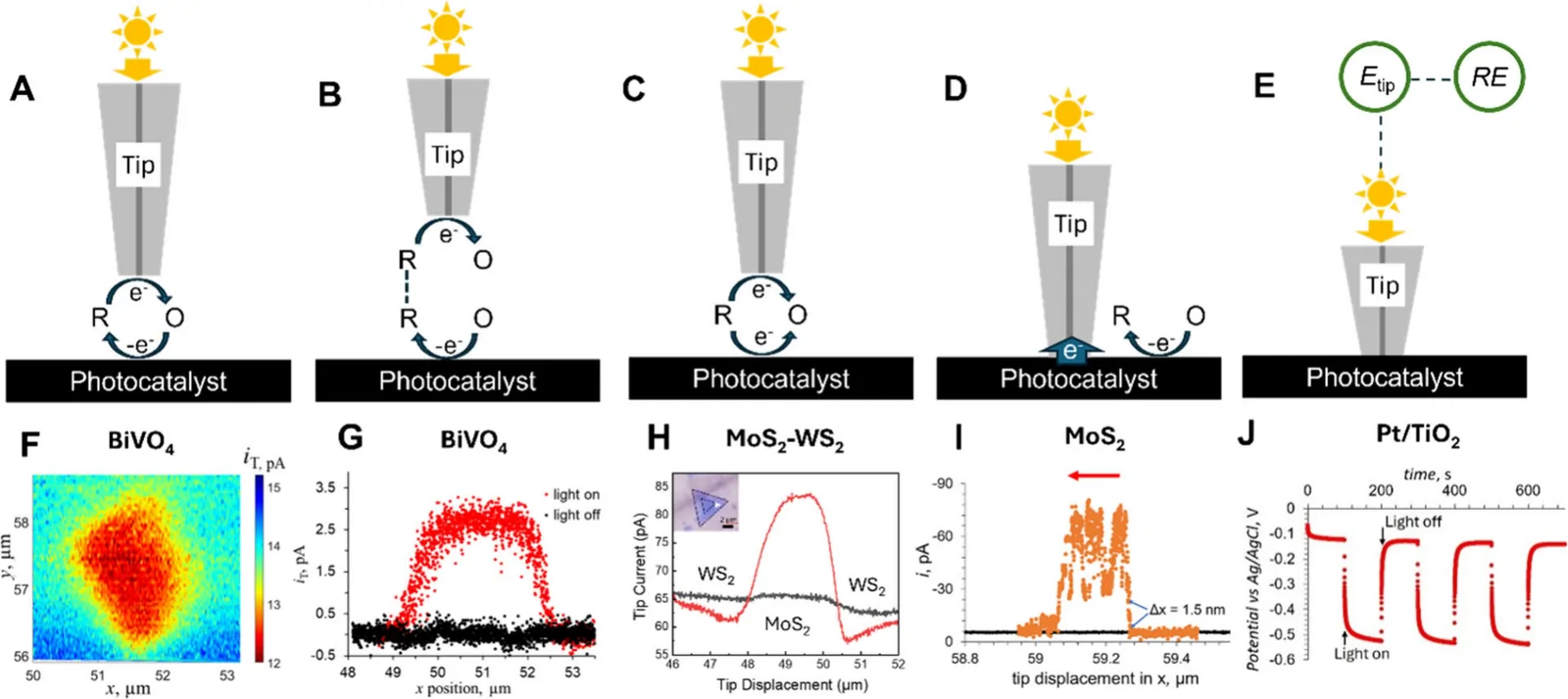
Schematic diagrams and representative experimental examples of five photo-SECM modes. (A, F) Feedback mode and corresponding 2D SECM topography image of a BiVO_4_ crystal attached to a glass surface, obtained with a Pt tip in 0.1 M K_2_SO_4_ containing 1 mM ferrocenemethanol (Fc). (B, G) SG–TC mode and a lateral scan recorded over a P:BiVO_4_ microcrystal showing detection of photogenerated H_2_ under illumination (red) relative to the dark state (black). The tip current is due to the hydrogen oxidation reaction (HOR). (C, H) Redox competition mode and lateral scans over a MoS_2_–WS_2_ heterojunction in the dark (black dots) and under illumination (red dots) in 1 mM Fc solution. The tip potential was *E*_T_ = 0.4 V vs Ag/AgCl. (D, I) Tunneling mode and lateral scans acquired over a MoS_2_ monolayer triangle on sapphire in the dark (black) and under illumination (orange) in 1 mM HClO_4_ + 0.1 M KCl. (E, J) Potentiometric mode and chopped-light potential transients obtained with the tip positioned within tunneling distance from a Pt nanoparticle electrodeposited on the TiO_2_ surface. Reproduced with permission from (F,G) ref 30, copyright 2023 American Chemical Society; (H) ref 1, copyright 2026 American Chemical Society; (I) ref 4, copyright 2025 American Chemical Society; and (J) ref 2, copyright 2026 National Academy of Sciences.

The SG–TC mode of photo-SECM (Figure 1B) directly measures local fluxes of reaction products generated at the photocatalyst surface, such as hydrogen (Figure 1G)^30^ or oxygen. It was applied to directly detect hydrogen and oxygen fluxes produced by overall water splitting (OWS) on individual Al:SrTiO_3_/Rh_2–y_Cr_y_O_3_ microcubes and confirm stoichiometric H_2_/O_2_ production.^29^ This approach was then extended to bias-dependent single-particle photoelectrochemistry by attaching an individual Al:SrTiO_3_/Rh_2–y_Cr_y_O_3_ cube to a carbon-fiber microelectrode (Figure 2A).^32^ The approach curves obtained for OER and HER at the same illuminated surface (Figure 2B, 2C) were fitted to the theoretical curves obtained by COMSOL simulations to determine the local O_2_ and H_2_ fluxes, which were found to follow the applied potential dependence of HER and OER measured on the same illuminated particle (Figure 2D).

**Figure 2:**
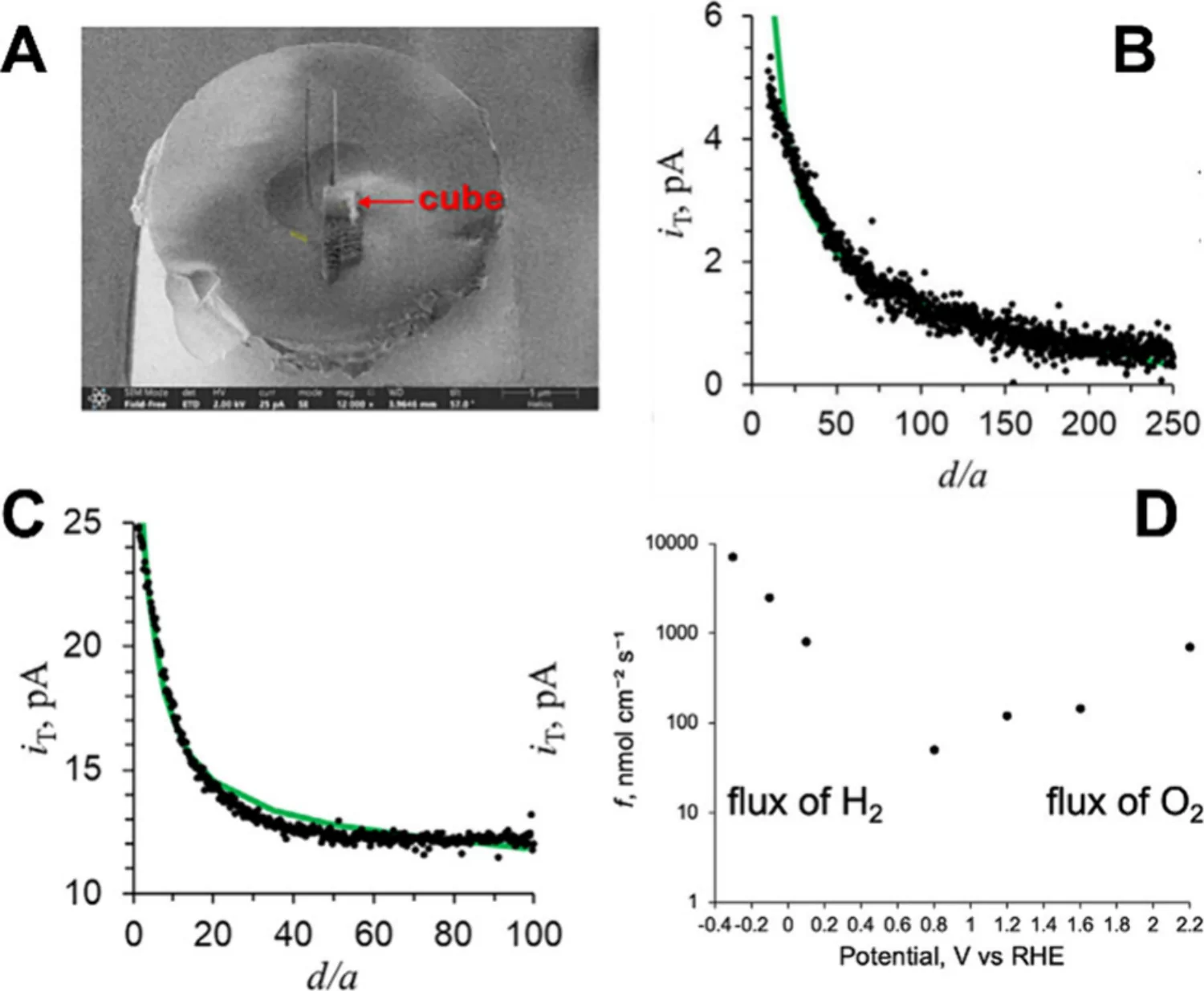
(A) SEM image of an Al:SrTiO_3_/Rh_2−y_Cr_y_O_3_ cube attached to a carbon-fiber microelectrode; the carbon fiber is surrounded by an insulating glass layer. (B, C) Experimental SECM approach curves (symbols) obtained with a Pt tip positioned over the center of the top face of the illuminated Al:SrTiO_3_/Rh_2−y_Cr_y_O_3_ cube and fitted to theoretical curves (solid lines). (B) The substrate potential was *E*_*S*_ = 1.6 V vs RHE, corresponding to OER and yielding the calculated O_2_ flux, *f*_O_2__ = 145 nmol cm^–2^ s^–1^. (C) *E*_*S*_ = 0.1 V vs RHE, corresponding to HER and yielding the calculated H_2_ flux, *f*_H_2__ = 0.8 nmol cm^–2^ s^–1^. (D) Potential dependence of HER and OER rates on a single illuminated Al:SrTiO_3_/Rh_2−y_Cr_y_O_3_ cube in 0.1 M K_2_SO_4_. Adapted with permission from ref 32, copyright 2025 Wiley.

The same general strategy was used to visualize heterogeneity on single P:BiVO_4_ microcrystals, where feedback-mode first identified and imaged the crystal, and SG–TC measurements revealed substantial spatial variations in local HER and OER activity across different regions of the same particle^30^ that could not be observed by ensemble averaged bulk measurements.

The above examples show that photo-SECM can measure interfacial charge-transfer kinetics of photo­(electro)­catalytic processes on the nanoscale. By contrast, measuring local surface potentials, band energetics, and driving forces for charge separation and interfacial charge transfer under *operando* conditions remains challenging. Techniques such as SPV,^36^ PL mapping,^37^ and PEEM^16^ have provided valuable insight into photogenerated charge redistribution and local electronic structure, but are generally performed in air or vacuum, while electrochemical AFM^38,39^ can probe local potentials in liquid but does not directly provide colocated measurements of photocatalytic product flux. Complementary fluorescence-based approaches have also revealed pronounced spatial heterogeneity in catalytic activity. Chen and co-workers combined single-molecule reaction imaging with photocurrent mapping to identify subparticle-scale active regions and determine how the placement of catalysts affects the performance of individual photoanodes.^17^ Tachikawa *et al.* used single-molecule fluorescence imaging and kinetic analysis to resolve crystal-face-dependent photocatalytic activity on individual anatase TiO_2_ particles.^40^ Hofkens and co-workers pioneered single-turnover fluorescence mapping to reveal crystal-face-dependent catalytic reactivity on individual catalyst crystals.^41^ These studies established the value of single-particle and single-molecule imaging for resolving local reaction heterogeneity.

In this Account, we discuss the development of new photo-SECM methodology that can correlate spatial activity maps to local energetics under *operando* conditions. The redox competition mode (Figure 1C) can be used for in situ imaging of photogenerated charge separation in two-dimensional (2D) photocatalysts (Figure 1H).^1^ The tunneling mode, in which the tip is brought within the tunneling distance from the sample surface (Figure 1D) enables ultrahigh-resolution mapping of photocatalytic activity and lateral charge transport (Figure 1I).^4^

Finally, the potentiometric mode (Figure 1E) measures the surface potential (Figure 1J) and local photovoltage indicated by differences in electrochemical potentials between electron accumulation and hole accumulation sites.^2^ After bringing the nanoprobe close to the sample surface in the tunneling mode, the same tip is then used for open-circuit potentiometric sensing at essentially the same nanoscale location. The sequential dual-mode amperometric/potentiometric photo-SECM enables *operando* measurement of both local electrochemical reaction flux and local electrochemical driving force at the same active site, thereby providing direct access to the local potential–rate relationship under catalytic turnover. In contrast to surface photovoltage or surface-potential-difference methods, which are typically performed in vacuum, air, or moist environments, the potentiometric signal in photo-SECM is referenced to the electrolyte redox scale and thus probes the steady-state surface energetics of working photocatalysts in solutions under operational conditions. It addresses the previous challenge of averaged potentiometric measurements^42^ using macroscopic photocatalyst-derived photoelectrodes. The electrolyte-referenced local surface potential is measured by the nanotip near the sample surface, and the light-induced change in its value represents surface photovoltage. Combined with redox potentials and kinetic overpotentials, this potential can be used to define the local electrochemical driving force for a given half-reaction with its thermodynamic redox potential. Table 1 compares different modes of photo-SECM operation discussed in this Account.

**Table 1:**
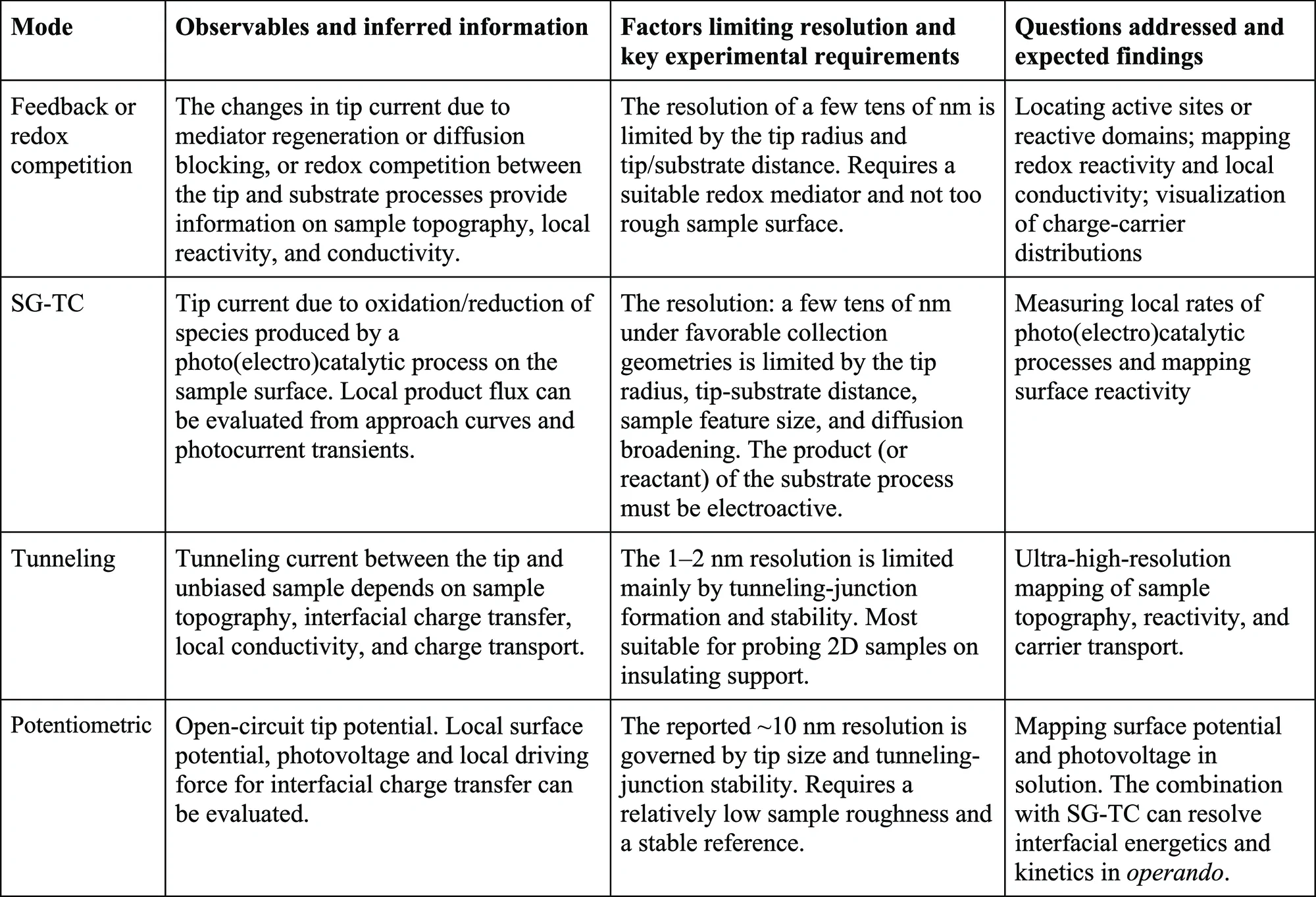
Summary of Photo-SECM Modes of Operation

Combining amperometry and potentiometry is powerful because it enables the *operando* measurements of electrochemical activity and driving force at the same nanoscale location with the same nanotip. These advances establish a multimodal photo-SECM platform that integrates topographic imaging, localization of catalytically active regions, measurements of photocatalytic kinetics, in situ visualization of photogenerated charge-carrier distributions, and direct mapping of local surface potentials and photovoltages, thereby enabling *operando* correlation of catalyst morphology, local reactivity, charge separation, and interfacial energetics in photocatalytic systems. This framework enables extraction of local rate constants and reconstruction of the coupling among catalyst geometry, electrostatic structure, and interfacial kinetics. In this way, multimodal photo-SECM can reveal built-in electric fields, defects, and heterojunctions and distinguish the contributions of charge-separation efficiency and catalytic surface kinetics.

## Imaging Charge Separation and Probing Driving Forces in 2D Photocatalysts

The combination of the feedback and redox competition modes of photo-SECM (Figure 1A,C) was recently employed to directly image the migration of photogenerated electrons and holes in two-dimensional semiconductors.^1,31^ High-resolution photoelectrochemical imaging of MoS_2_ triangles on SiO_2_ support (Figure 3) produced the first evidence of charge separation at the semiconductor/insulator interface.^31^ The ferrocenemethanol mediator was oxidized at the tip to generate Fc^+^, and the illuminated sample surface either reduced Fc^+^ (positive feedback; Figure 3Ai) or oxidized Fc (redox competition; Figure 3Aii). The former process occurred on the MoS_2_ triangle, where photogenerated electrons accumulate, and resulted in the increased *i*_T_, while photogenerated holes that migrated to the portion of SiO_2_ surface adjacent to the edge of the triangle and oxidized Fc caused the decrease in *i*_T_ below the negative-feedback level (Figure 3C). By selectively identifying electron-rich and hole-rich regions, this approach enables simultaneous imaging of spatial distributions of photogenerated electrons and holes in the photocatalyst under *operando* conditions. The corresponding line scan further showed that the competition response extended laterally over micrometer distances on the SiO_2_ surface, indicating that photogenerated holes migrated from MoS_2_ onto the insulating support (Figure 3D).

**Figure 3:**
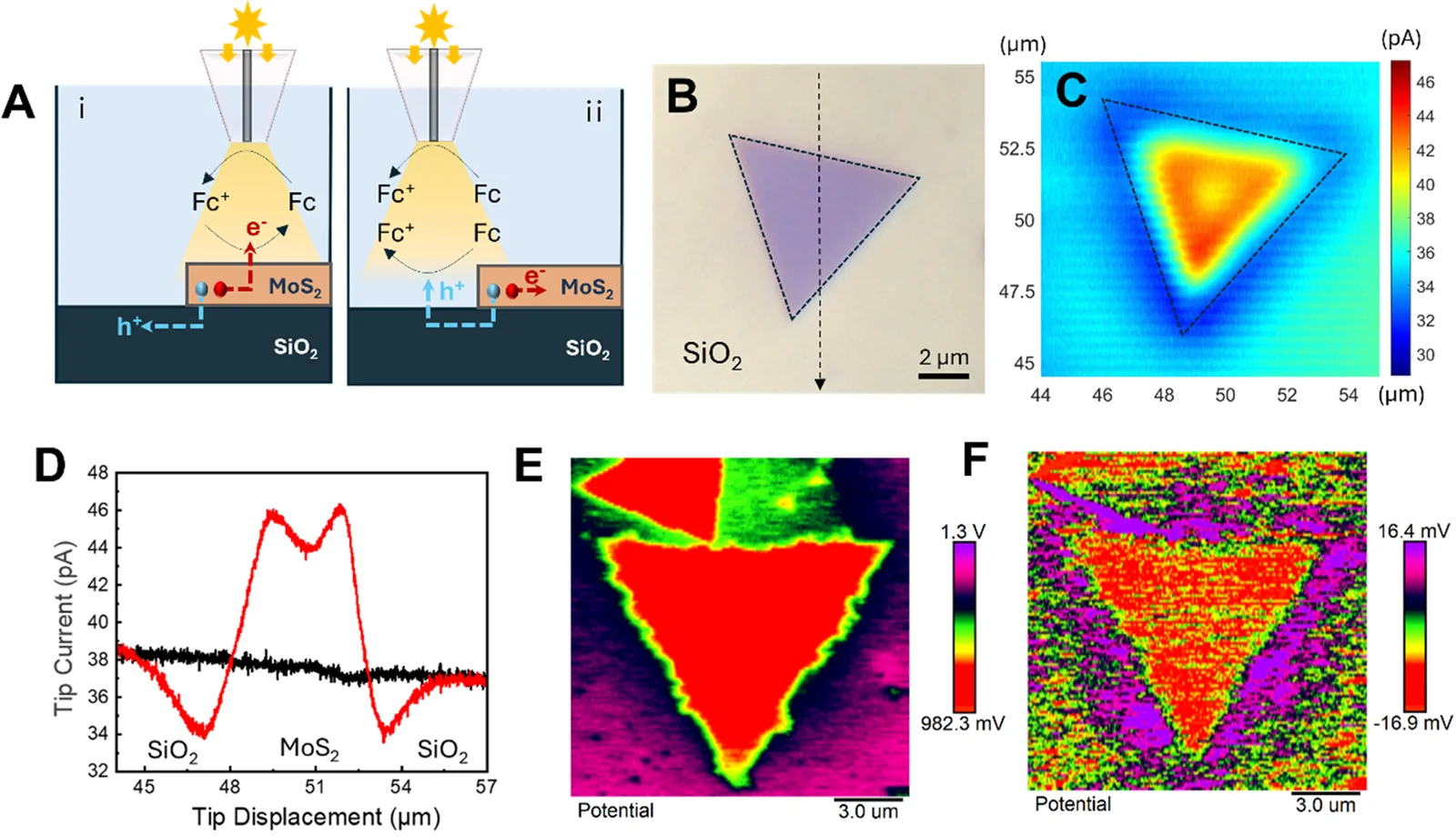
Photo-SECM and SPV of a MoS_2_ triangle. (A) Schematic representation of the positive SECM feedback over the illuminated MoS_2_ surface (i) and redox competition over SiO_2_ surface (ii). (B) Optical micrograph of a 5L MoS_2_ triangle. (C) Constant-height photo-SECM image of the same triangle. Solution contained 1 mM Fc in 0.1 M phosphate buffer (pH 7). *E*_T_ = 0.4 V vs Ag/AgCl. *a* = 150 nm. A 250 W HgXe lamp provided broadband illumination with an irradiance of ∼2 mW cm^–2^ and a spot size of several tens of micrometers. (D) Lateral scans along the specified trajectory (black line in panel B) under illumination (red curve) and in the dark (black). Dark-state potential (E) and SPV (F) images of a 4L MoS_2_ triangle on SiO_2_ support in the air. Adapted from ref 31, copyright 2025 American Chemical Society.

To elucidate the driving force underlying this behavior, liquid-phase photo-SECM measurements were complemented by Kelvin probe force microscopy (KPFM) and SPV mapping in air. The dark-state KPFM image (Figure 3E) showed a gradual potential change across the SiO_2_–MoS_2_ boundary, from which a lateral built-in electric field of about 1.7 kV cm^–1^ was estimated. This interfacial field provides the driving force for photogenerated carrier separation, driving holes laterally onto SiO_2_ while leaving electrons on MoS_2_. This picture is directly supported by the SPV image under illumination (Figure 3F), showing positive photovoltage on SiO_2_ and negative photovoltage on MoS_2_. Together, these results demonstrate that insulating support can effectively facilitate separation of electrons and holes in semiconductors.

While the combination of photo-SECM with KPFM/SPV can visualize the carrier distribution and the direction of the built-in field, it does not provide the band structure information needed for analysis of charge transfer or to image and interpret interfacial recombination processes. A more complete mechanistic picture of charge-separation and recombination in monolayer in-plane MoS_2_–WS_2_ heterojunctions was obtained by combining photo-SECM and SPVM with photoluminescence (PL) imaging, and ultraviolet photoelectron spectroscopy (UPS).^1^ An optical image of the heterojunction and the corresponding constant-height photo-SECM map (Figure 4A,B) show positive feedback over the MoS_2_ domain and a redox-competition response over the adjacent WS_2_ region, corresponding to the accumulation of photogenerated electrons in MoS_2_ and holes in WS_2_. This domain-selective charge separation is independently confirmed by SPVM (Figure 4C), which also showed that the lateral heterojunction generated a much larger photovoltage contrast than either the individual monolayers or a vertical MoS_2_–WS_2_ heterojunction. PL images (Figure 4D) added a critical element by revealing enhanced emission localized at the interface, indicating that the phase boundary also acts as a recombination center.

**Figure 4:**
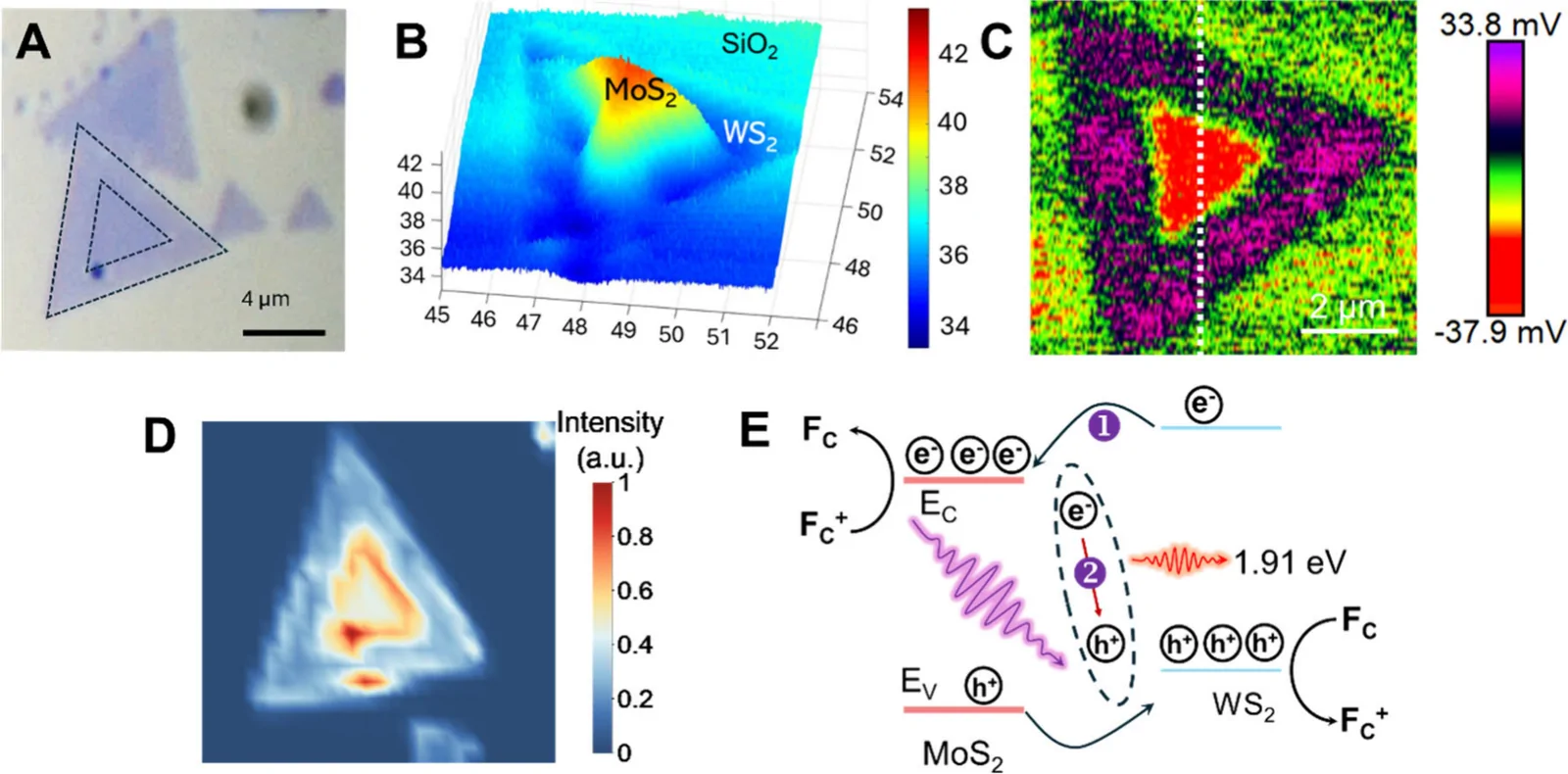
Optical micrograph (A), constant-height photo-SECM image (B), SPVM image (C), and PL image (D) of in-plane WS_2_–MoS_2_ heterojunctions. SPVM was performed with 450 nm illumination at approximately 14.9 mW and a spot size of about 1 mm; PL spectra and maps were acquired with a 532 nm laser at 1 mW and an excitation area of approximately 1 μm^2^. (E) Proposed energy band diagram of the heterojunction. The arrows represent electron transfer toward MoS_2_, hole transfer toward WS_2_, and the 1.91 eV interfacial radiative-recombination pathway. Adapted from ref 1, copyright 2026 American Chemical Society.

UPS provided the band offsets needed to construct the type-II energy diagram (Figure 4E), which explains why electrons migrate toward MoS_2_ and holes toward WS_2_. Taken together, these measurements showed that the monolayer in-plane MoS_2_–WS_2_ heterojunction supports efficient directional charge separation driven by intrinsic band alignment, while the same interface simultaneously introduces a localized recombination pathway.

The above examples show that in addition to photocatalytic activity and carrier distribution maps, the information about band structure, interfacial electric fields, and recombination pathways is required for understanding charge transfer processes in 2D photocatalysts. However, the interfacial energetics are typically measured in air or vacuum rather than under *operando* conditions. This limitation motivates the development of new photo-SECM modes for correlative nanoscale interrogation of interfacial energetics and charge-transfer kinetics in solutions.

## Tunneling Mode of Photo-SECM

The next step toward correlative imaging of surface kinetics and energetics of photocatalysts under *operando* conditions was the development of the tunneling mode of photo-SECM (Figure 1D,I).^4^ In a tunneling SECM experiment,^43^ a nanoelectrode is brought within ∼1 nm from the sample surface to form a tunneling junction (Figure 1D). At such short separation distances, a conductive specimen (e.g., a metal nanoparticle; NP) acts as a part of the nanotip, so that the current flowing at such a sample can be measured without attaching it to the tip surface. The tip applies an electric bias to the sample surface, and the charge-transfer process occurring at the photocatalyst/solution interface is the source of the tunneling current that flows between the tip and the photocatalyst (Figure 1D, I).

The transition from diffusion-based SECM feedback to tunneling can be observed by comparing the approach curves obtained in the dark ­(Figure 5A, black curve) and under illumination (Figure 5A, orange curve) in a 0.1 M HClO_4_ solution. In the dark, *i*_T_ produced by proton reduction decreases monotonically as the Pt tip approaches a MoS_2_ triangle, showing negative feedback. The approach curve under illumination (Figure 5A, orange curve) is indistinguishable from that obtained in the dark until the onset of the tunneling current occurs at a separation distance of a few nanometers. The magnitude of the photocurrent produced by the HER at the illuminated MoS_2_ surface can be evaluated from the chopped-light transients (Figure 5B). These transients recorded with the same tip touching the MoS_2_ triangle show the photocurrent a few tens of pA. This current is orders of magnitude smaller than the diffusion-limited current expected for a triangle with ∼10 μm side length, suggesting that the measured response is limited by the low steady-state photocarrier concentration in MoS_2_ and/or slow lateral transport of carriers in the triangle. Because carrier generation and in-plane transport are convoluted with interfacial charge transfer to the solution redox couple, disentangling these processes will require extending tunneling-mode photo-SECM to time-resolved measurements capable of probing carrier dynamics and interfacial charge-transfer kinetics on the μs–s catalytic time scale.

**Figure 5:**
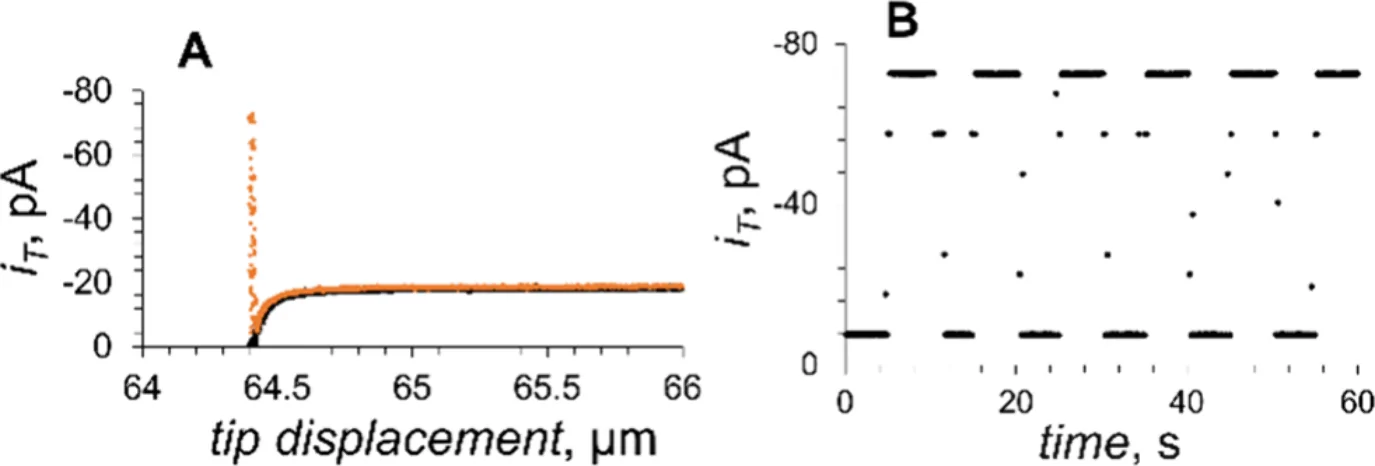
HER-based approach curves and chopped-light transients obtained over a MoS_2_ CVD-grown triangle attached to the sapphire surface. (A) Approach curves obtained in the dark (black curve) and under illumination (orange curve) with the same *a* = 59 nm Pt tip approaching the same spot on the MoS_2_ surface. (B) Tunneling mode chopped-light current transients recorded with the same tip touching the sample surface. Solution contained 1 mM HClO_4_ in 0.1 M KCl. *E*_T_ = −0.7 V vs Ag/AgCl. Adapted from ref 4, copyright 2025 American Chemical Society.

The spatial resolution of tunneling mode photo-SECM imaging is much higher than that attainable in diffusion-based SECM experiments. For instance, a lateral scan across an illuminated MoS_2_ monolayer triangle (orange symbols in Figure 1I) captured sharp changes in tunneling current with a lateral resolution of ∼1 nm, enabling direct visualization of hydrogen evolution sites. In conventional feedback mode imaging, the spatial resolution is largely determined by the tip radius and limited by mediator diffusion in the tip/substrate gap. Thus, the lateral scan over a mixed-phase MoS_2_ nanosheet ­(Figure 6A) shows relatively broad current transitions between the sapphire support and different MoS_2_ domains. In contrast, the tunneling-mode scan of a similar mixed-phase nanosheet (Figure 6B) exhibits much sharper boundaries and clearly resolves a narrow (∼50 nm wide) 2H region located between two 1T′ domains. A tunneling mode image (Figure 6C) reveals nanoscale heterogeneity, clearly distinguishing between single layer MoS_2_ triangles and a bilayer formed by two overlapping monolayer triangles based on the differences in the rate of lateral charge transport.

**Figure 6:**
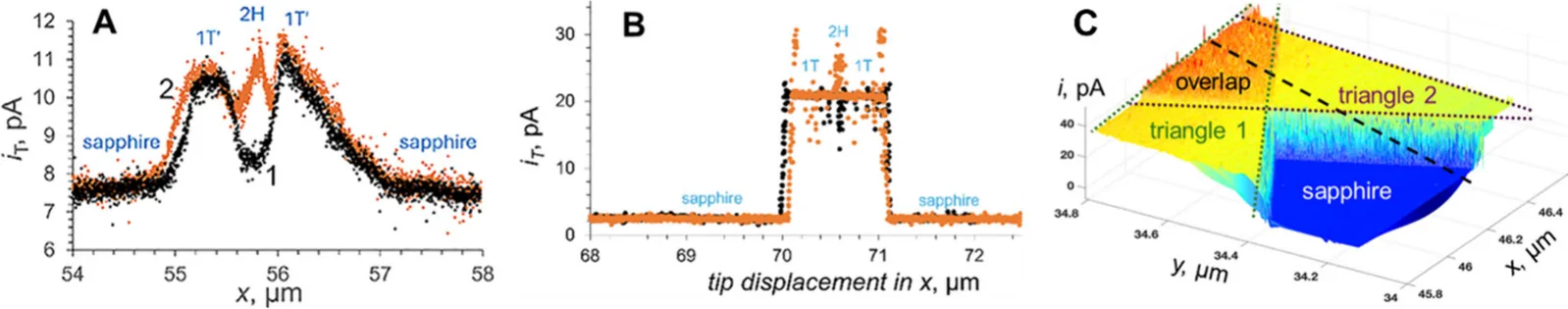
(A) Feedback and (B) tunneling mode lateral scans over mixed-phase MoS_2_ nanosheets in the dark (black) and under illumination (orange). Solution contained 1 mM K_4_Fe­(CN)_6_ and 0.1 M KCl. Tip radius, *a* = 45 nm. Tip potential, *E*_T_ = 0.4 V vs Ag/AgCl. (C) Colored 3D tunneling photo-SECM image with green and purple dotted lines showing the areas of two MoS_2_ triangles and the orange region corresponding to their overlap. The current was due to the oxidation of 1 mM Fc in 0.1 M KCl at the 45 nm Pt tip. Adapted from ref 4, copyright 2025 American Chemical Society.

A biased metal nanoelectrode placed within the tunneling distance from the photocatalyst surface is not a passive probe. It may perturb local electric fields, induce additional band bending and changes in light distribution. However, the anticipated tip-induced effects should be significantly lower than in STM, where the bias is applied between the probe and the sample, and not larger than in other established SPM techniques, such as conductive AFM. The electrical double layers at the tip and photocatalyst are expected to overlap, and the applied potential is partitioned across the tip compact layer, the gap, and the semiconductor/electrolyte interface. The quantitative modeling of this system is challenging because the tunneling junction cannot be visualized, and the exact separation distance is unknown.

In addition to increased spatial resolution, the tunneling mode of SECM allows one to establish a stable tunneling junction at the semiconductor/liquid interface by bringing the tip within ∼1 nm distance from the photocatalyst surface (Figure 5A). By switching the SECM from amperometric to potentiometric regime, local surface potentials and photovoltages can be mapped in solution.

## Amperometric/Potentiometric Photo-SECM of Nanostructured Photocatalysts

A correlative amperometric/potentiometric photo-SECM experiment typically begins with feedback-mode imaging of the sample to locate the photocatalyst surface and identify the domains of interest (Figure 1A). The SECM is then operated in SG/TC mode to map and/or quantify local photocatalytic product fluxes, e.g., H_2_ or O_2_ (Figure 1B). Subsequently, the tip can be brought within the tunneling distance from the sample surface (Figure 1D), and the instrument can be switched to potentiometry (Figure 1E) to measure local surface potentials of a selected photocatalyst or cocatalyst domain under *operando* conditions.

One application of this approach is to map oxygen evolution kinetics and energetics during OWS on a Nb-doped TiO_2_ rutile (110) single crystal with Pt NPs electrodeposited on its surface (Pt/Nb:TiO_2_), as shown in Figure 7. A feedback-mode topography image (Figure 7A) was used to locate a ∼100 nm Pt NP and define the trajectory of the tip line scan across the Pt/TiO_2_ boundary (dotted line in Figure 7A). The tip was biased at −0.7 V vs Ag/AgCl to collect O_2_ produced at the sample surface under illumination. The resulting line scan (Figure 7B) shows that the strongest O_2_ signal appears at location 1 (*x* = 52.90 μm corresponding to the red symbol in Figure 7A) over the TiO_2_ surface about 100 nm away from the Pt edge. The much lower ORR current at the tip was recorded either closer to the Pt NP or further away from it, or above the particle surface.

**Figure 7:**
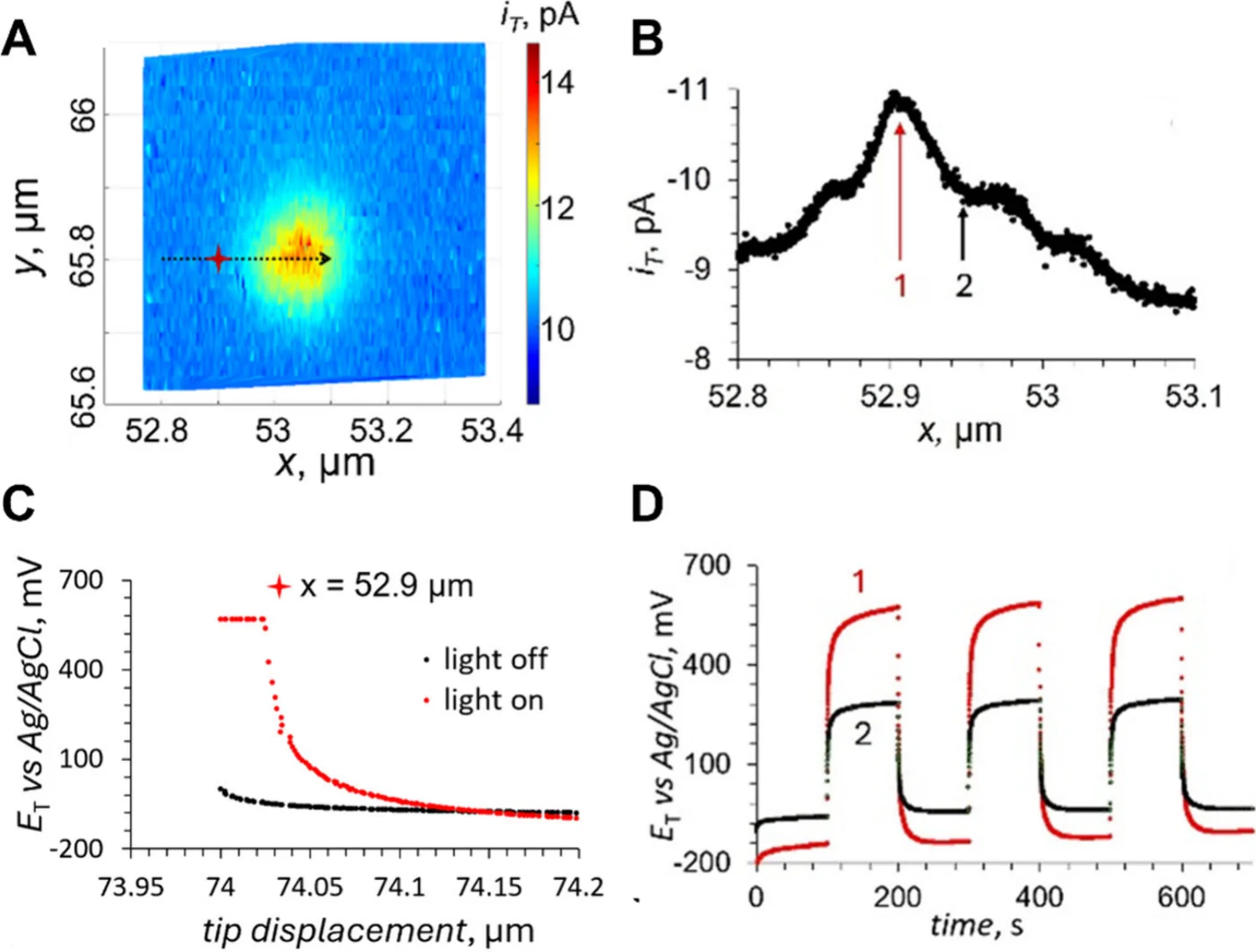
Amperometric and potentiometric photo-SECM characterization of the OER-active region near a Pt NP on TiO_2_. (A) Feedback-mode topography image of a Pt NP on the TiO_2_ surface. (B) Lateral scan recorded under illumination along the trajectory indicated by the dotted line in panel A with the tip biased negatively to detect O_2_. (C) Potentiometric approach curves recorded under illumination (red) and in the dark (black) over location 1 on the TiO_2_ surface (*x* = 52.90 μm; red symbol in A). (D) Chopped-light potential transients obtained with the same tip positioned within the tunneling distance from the TiO_2_ surface at locations 1 (red curve) and 2 (black curve; *x* = 52.95 μm in B). Solution contained 1 mM K_4_Fe­(CN)_6_ and 10 μM K_3_Fe­(CN)_6_ in 0.1 M KCl (A) and 0.1 M PB with pH 7 (B–D). Tip radius, *a* = 30 nm. *E*_T_, V vs Ag/AgCl = 0.4 (A) and −0.7 (B). Adapted with permission from ref 2, copyright 2026 National Academy of Sciences.

The local surface potentials were measured to investigate the origins of the hot spot for water oxidation visualized in Figure 7B. When the same SECM tip approached the TiO_2_ surface at location 1 in the dark (black curve in Figure 7C), *E*_T_ at the surface was only slightly different from its bulk value. By contrast, under illumination, *E*_T_ changed from about −100 mV in bulk solution to ∼600 mV vs Ag/AgCl at the TiO_2_ surface (red curve in Figure 7C). The latter value, which is sufficiently positive for OER, was confirmed by chopped-light potential transients recorded at location 1 after bringing the tip within the tunneling distance from the TiO_2_ surface (red curve in Figure 7D), whereas the much lower surface potential at location 2 (black curve in Figure 7D) does not provide sufficient driving force for water oxidation. These observations can be understood in terms of transport and accumulation behaviors of photogenerated holes in Nb:TiO_2_, which possess limited mobility, with diffusion lengths typically around 10 nm, and therefore accumulate near Pt NPs, where oxidized Pt domains provide favorable sites for hole trapping and oxidation. Although the measured trends and reactivity maps are generally reproducible, quantitative reproducibility of photo-SECM experiments at the nanoscale is challenging because the methodologies for fabricating identical nanotips and repeated precise tip positioning over the same location are currently unavailable.

A similar approach was used to investigate OWS on the geometry-programmed GaInP/GaN/IrO_*x*_/Rh–CrO_*x*_ photocatalyst, which showed high solar-to-hydrogen (STH) efficiency of unassisted OWS for a single narrow-bandgap (1.84 eV) GaInP light absorber. This efficiency is achieved by laterally arranging oxidative (IrO_*x*_) and reductive (Rh–CrO_*x*_) cocatalyst domains, as probed by amperometric/potentiometric photo-SECM (Figure 8). After locating the cocatalyst domains by feedback-mode SECM, SG/TC approach curves were recorded under illumination to detect O_2_ generated on IrOx (Figures 8A) and H_2_ generated on Rh–CrOx (Figure 8B). The approach curves were fitted with COMSOL mass-transport simulations, yielding local O_2_ and H_2_ fluxes of 1.5 μmol cm^–2^ s^–1^ and 8.0 μmol cm^–2^ s^–1^, respectively, under OWS conditions.

**Figure 8:**
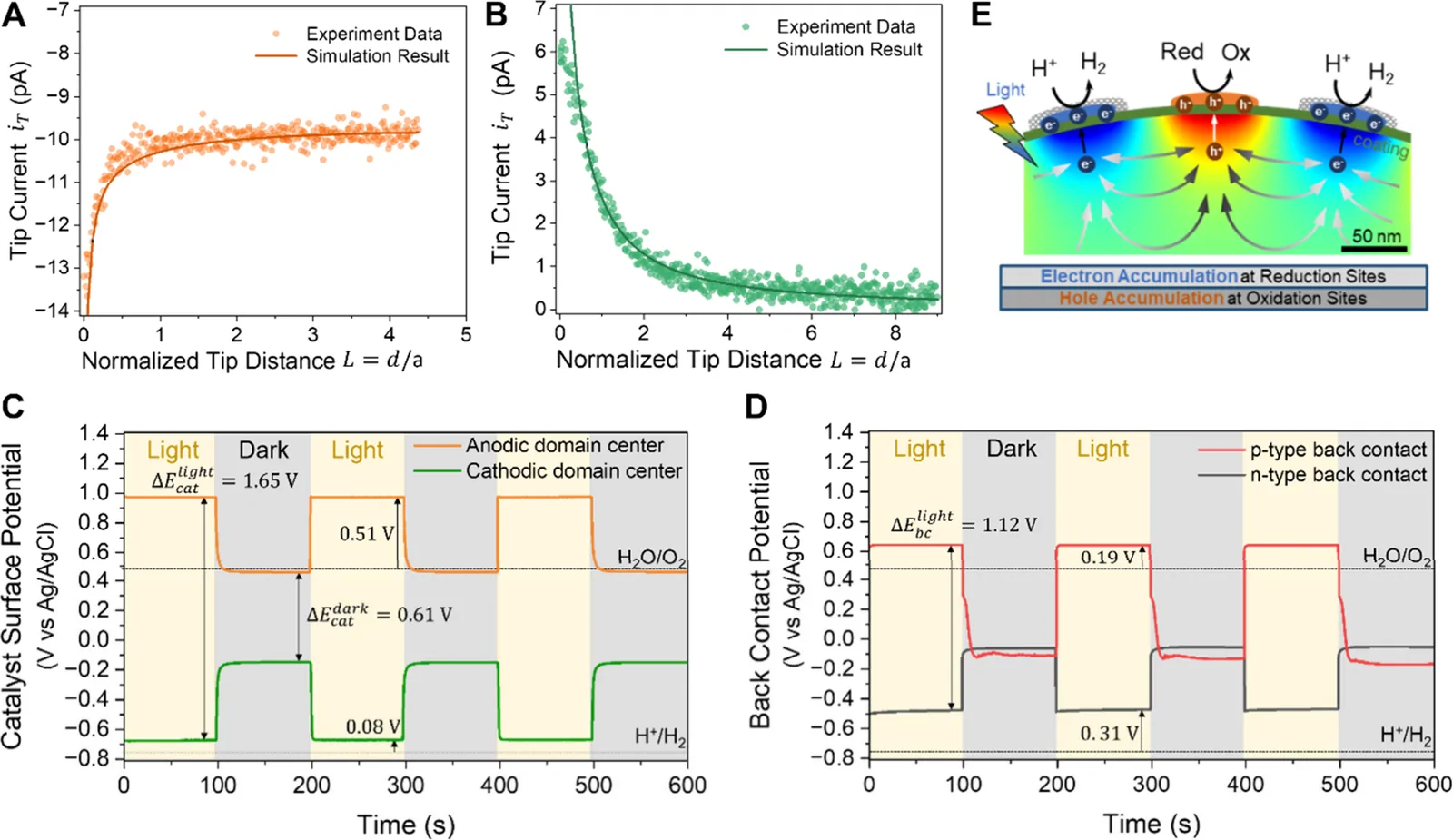
Photo-SECM measurements of OWS kinetics and surface energetics of GaInP/GaN/pattern-IrO_*x*_/Rh-CrO_*x*_ photocatalyst. Corresponding approach curves for oxygen from IrO_*x*_ (A) and hydrogen from Rh–CrO_*x*_ (B) collection under illumination. Local OER and HER fluxes are subsequently obtained by fitting these approach curves with a COMSOL transport model. Chopped-light potentiometric tip transients (C) and back-contact potential transients for samples with n-type and p-type ohmic contacts (D). (E) Potential profile generated by COMSOL semiconductor model using locally probed potential at reductive and oxidative sites, adapted with permission from ref 44, copyright 2021 National Academy of Sciences.

The same nanotip was then used to obtain chopped-light potential transients at the surfaces of either Rh–CrO_*x*_ (orange curve in Figure 8C) or the neighboring IrO_*x*_ site (green curve in Figure 8C) separated by ∼400 nm. The data show that the anodic and cathodic domains developed an *operando* surface-potential difference of ∼1.7 V under illumination, whereas the back-contact potential splitting measured using p-type and n-type contacts was only about 1.1 V (Figure 8D). This comparison shows that the local interfacial electrochemical potential difference between the oxidative and reductive domains in operando, which enables efficient OWS despite the limited bulk photovoltage of GaInP, cannot be inferred solely from the back-contact quasi-Fermi-level splitting. Nanoscale *operando* measurements and modeling are required to clarify photocatalytic charge separation. Area-averaged open-circuit measurements, ensemble spectroscopies, potential-sensing probes, surface photovoltage methods, and self-consistent simulations have all provided valuable views of photocatalyst energetics and carrier separation.^37,42,45–48^ Amperometric/potentiometric photo-SECM adds a more local view: the current response, product flux, and surface potential can be measured from the same nanoscale region. This is especially useful for photocatalysts in which H_2_ and O_2_ evolution sites are close together, because local reaction rates and local driving forces vary over submicron distances and are difficult to separate in spatially averaged measurements.

The photo-SECM measurements of local HER or OER rates and surface potentials at active sites provide experimental boundary conditions for mass-transport and semiconductor charge-transport models. A previously developed mathematical model of charge separation and directional transport of photogenerated carriers between the cathodic and anodic sites of a photocatalyst coupled with interfacial charge-transfer processes is shown schematically in Figure 8E.^44^ Specifically, the model should account for carrier generation, carrier transport/recombination, spatial carrier distribution, band-edge energetics and dielectric response at surfaces, and interfacial charge-transfer boundary conditions. Parameters such as doping concentration, dielectric constant, carrier mobility or diffusion length, electron and hole carrier lifetime, in-gap state distribution, band-edge energetics, surface recombination rates, and interfacial charge-transfer rate law and rate constants are needed. Essentially, modeling and photo-SECM data analysis require these parameters to determine the relationship between local surface potential, band bending, carrier availability, and measured reaction flux. This analysis helps explain how photogenerated carriers are separated, transported to different surface sites, and used to drive HER and OER under operating conditions. It also links the local surface potential at each site to the driving force available for the corresponding half-reaction.^44,49,50^ The calculated cross-sectional potential and electron/hole concentration profiles show that charge-carrier separation is sustained by a combined energetic and kinetic asymmetry at the interfacial domains.

The best photocatalysts need to extract all photogenerated electrons and holes, which elucidates the photocatalyst design principle. Figure 8E shows that within the depth of light absorption (e.g., 100 nm), photogenerated electrons and holes can be transported to their respective electron and hole accumulation sites, suggesting that beyond the, e.g., 100 nm, depth in photocatalysts, bulk carrier recombination can build up a potential difference. That difference was experimentally manifested as a potential difference between the back and front contacts of a model photocatalyst system, which can be probed by photo-SECM (e.g., the orange curves in Figure 8C vs [Fig fig8]D for electron potential variations). So is the case for surface vs bulk potentials for holes, e.g., the green curves in Figure 8C vs [Fig fig8]D for hole potential variations.

Thus, the data in Figure 8 show why the back-contact or spatially averaged measurements cannot, by themselves, define the local interfacial energetics that drive photocatalysis. Back-contact photovoltage or quasi-Fermi-level splitting reports the potential at photocatalyst centers, whereas potentiometric photo-SECM probes the local surface potential at the specific cocatalyst/electrolyte interface where charge transfer occurs. These quantities can differ because local band bending, cocatalyst charging, double-layer potential drop, and interfacial kinetic losses are spatially heterogeneous and are not fully captured by the back contact. Thus, macroscopic photocurrent, onset potential, and back-contact photovoltage should be viewed as spatially averaged or contact-projected outcomes of heterogeneous local flux and driving-force distributions, which multimodal photo-SECM can resolve directly.

Potentiometric photo-SECM experiments are only feasible if the nanoelectrode probe can make physical contact with the sample, and charge carriers equilibrate between the surface and the tip, enabling direct measurement of the local surface potential. Very rough or poorly conductive materials, with extremely low steady-state carrier densities are not suitable for such measurements. For instance, no reliable surface potential value can be extracted from the dark-condition approach curve (black curve in Figure 7C), even though the tip was brought within the tunneling distance from the TiO_2_ surface.

## Conclusion and Outlook

The recent rapid progress in nanoscale photo-SECM has opened new avenues for the study of photo­(electro)­catalysts. Our recent results and those reported by others suggest that no single technique can fully resolve the coupled processes of light absorption, charge separation, carrier transport, interfacial charge transfer, and photocatalytic activity that determine the performance of nanostructured photocatalysts. One can elucidate some of those processes by combining photo-SECM with complementary SPM approaches such as surface photovoltage microscopy, potential-sensing methods, and photoconductive AFM. However, those techniques typically operate under vacuum or in air and cannot be used in conjunction with photo-SECM to address the same nanoscale surface domain. Using a multimodal SECM approach that combines surface potentiometry with SG/TC-based flux measurements and/or feedback-mode imaging, one can directly map the steady-state energetic landscape along with the spatial distribution of reaction rates on individual catalytic domains under *operando* conditions. Measuring local activity and local driving force within the same microscopic sample area enables the distinction of the contributions of various factors to photocatalyst performance. Combined with transport and kinetic modeling, this approach should enable more direct correlations between local activity, driving force, and materials properties and better understanding of local photoelectrochemical behavior, which is essential for guiding the design of efficient and selective photo­(electro)­catalysts.

A near-term opportunity is to move multimodal photo-SECM from a manually executed, point-by-point characterization method to a more automated, statistically robust mapping platform. Such near-term automation could focus on the most time-consuming step to improve statistics. By repeating the sequential amperometric–potentiometric protocol over a predefined x–y grid, photo-SECM can transform single-site measurements into operando maps of local product flux and surface potential at dynamic solid–liquid interfaces, resolving nanoscale heterogeneity in charge separation, quasi-Fermi-level distributions, and interfacial reaction kinetics. Other immediate improvements can include a fine tip approach, automated tip positioning, approach-curve analysis, real-time screening, and drift correction during area scans. Such automation can help identify heterogeneous “hot spots” that should be ubiquitous but may be missed otherwise in manually selected regions.

A longer-term goal is to integrate automated photo-SECM with AI-assisted image recognition. For example, the signal intensity and distribution of multiple heterogeneous “hot spots” can provide valuable insights into coupled charge-transfer kinetics, local transport, and chemical environments. At present, photo-SECM primarily quantifies redox active products that reach the probe tip. (Although the possibility of quantification of surface-adsorbed species and unstable transient species has been demonstrated.^51^) Spatial integration of these local fluxes and generation rates over many domains or particles can then yield an ensemble internal quantum efficiency for the photocatalyst system; comparison with the incident rather than absorbed photon flux would instead provide an external or apparent quantum efficiency. Thus, photo-SECM does not directly provide quantum efficiency from the tip current alone, but its combination with local potentiometry, optical modeling, and semiconductor charge-transport analysis offers a pathway from nanoscale current measurements to spatially resolved carrier-collection efficiencies and, ultimately, the ensemble quantum efficiency of a heterogeneous photocatalyst. The potential sensing mode operates under a tunneling mechanism, which ensures high spatial resolution compared to current flux and approaching curve methods, but it still does not resolve redox-inactive adsorbates or short-lived intermediates. On this account, multimodal correlative SECM, including substrate generation/tip collection mode and local potential sensing reviewed herein, can collectively address quantification of surface-adsorbed species and transient species. To summarize, real-time approach-curve analysis, multiphysics continuum modeling, and physics-informed machine learning framework can be combined to help decouple charge separation performance from catalytic surface kinetics and semiconductor transport behavior. This AI-assisted direction should be viewed not as a replacement for advanced spectroscopy or physical modeling, but as a complementary route toward statistically grounded, predictive understanding of photocatalyst operation. It aims to reveal how band energetics, built-in electric fields, surface defects, cocatalyst domains, and electrolyte microenvironments jointly drive light-induced chemical kinetics and redox selectivity.^37^

## References

[1] Huang Q., Wang Z., Liu R., Yao H., Ni C., Bo T., Wu S., Sun F., Fan F., Mirkin M. V. (2026). In Situ
Imaging Reveals Efficient Charge Separation in Monolayer MoS_2_-WS_2_ Type-II Heterojunctions. J.
Am. Chem. Soc..

[2] Bo T., Su H., Wang Z., Bae J. H., Askarova G., Hu S., Mirkin M. V. (2026). Probing charge-transfer processes in Pt/TiO_2_ photocatalysts by amperometric/potentiometric photo-SECM. Proc. Natl. Acad. Sci. U.S.A..

[3] Ma X., Li Z., Amirifard M., Ennis Q., Su H., Shang J., Zhang W., Yanagi R., Lee Y., Kortshagen U., Hu S. (2026). Enhanced Photocatalytic Charge Separation in Coating-Protected Porous
Silicon Quantum Dot Films With Stability Approaching 100 h. Adv. Funct. Mater..

[4] Bo T., Ghoshal D., Wilder L. M., Miller E. M., Mirkin M. V. (2025). High-Resolution
Mapping of Photocatalytic Activity by Diffusion-Based and Tunneling
Modes of Photo-Scanning Electrochemical Microscopy. ACS Nano.

[5] Nishioka S., Osterloh F. E., Wang X., Mallouk T. E., Maeda K. (2023). Photocatalytic
water splitting. Nat. Rev. Methods Primers.

[6] Fang S., Rahaman M., Bharti J., Reisner E., Robert M., Ozin G. A., Hu Y. H. (2023). Photocatalytic
CO_2_ Reduction. Nat. Rev. Methods
Primers.

[7] Wang C. C., Li J. R., Lv X. L., Zhang Y. Q., Guo G. (2014). Photocatalytic
Organic Pollutants Degradation in Metal–Organic Frameworks. Energy Environ. Sci..

[8] Walter M. G., Warren E. L., McKone J. R., Boettcher S. W., Mi Q., Santori E. A., Lewis N. S. (2010). Solar Water Splitting Cells. Chem. Rev..

[9] Zhang L., Liardet L., Luo J., Ren D., Grätzel M., Hu X. (2019). Photoelectrocatalytic arene C–H amination. Nat. Catal..

[10] Zhang Z., Yates J. T. (2012). Band bending
in semiconductors: chemical
and physical consequences at surfaces and interfaces. Chem. Rev..

[11] Sivula K., Van De Krol R. (2016). Semiconducting materials for photoelectrochemical energy
conversion. Nat. Rev. Mater..

[12] Corby S., Rao R. R., Steier L., Durrant J. R. (2021). The Kinetics of
Metal Oxide Photoanodes from Charge Generation to Catalysis. Nat. Rev. Mater..

[13] Cowan A. J., Durrant J. R. (2013). Long-Lived Charge Separated States
in Nanostructured
Semiconductor Photoelectrodes for the Production of Solar Fuels. Chem. Soc. Rev..

[14] Ding C., Shi J., Wang Z., Li C. (2017). Photoelectrocatalytic Water Splitting:
Significance of Cocatalysts, Electrolyte, and Interfaces. ACS Catal..

[15] Li Q., Ni C., Cui J., Li C., Fan F. (2025). Impact of
Reaction
Environment on Photogenerated Charge Transfer Demonstrated by Sequential
Imaging. J. Am. Chem. Soc..

[16] Chen R., Ren Z., Liang Y., Zhang G., Dittrich T., Liu R., Liu Y., Zhao Y., Pang S., An H., Ni C., Zhou P., Han K., Fan F., Li C. (2022). Spatiotemporal
imaging of charge transfer in photocatalyst particles. Nature.

[17] Sambur J. B., Chen T.-Y., Choudhary E., Chen G., Nissen E. J., Thomas E. M., Zou N., Chen P. (2016). Sub-particle reaction
and photocurrent mapping to optimize catalyst-modified photoanodes. Nature.

[18] Huang T.-X., Dong B., Filbrun S. L., Okmi A. A., Cheng X., Yang M., Mansour N., Lei S., Fang N. (2021). Single-molecule
photocatalytic dynamics at individual defects in two-dimensional layered
materials. Sci. Adv..

[19] Mao X., Chen P. (2022). Inter-Facet Junction Effects on Particulate Photoelectrodes. Nat. Mater..

[20] Zhang J., Wang X., Wang X., Li C. (2025). Heterophase
Junction
Effect on Photogenerated Charge Separation in Photocatalysis and Photoelectrocatalysis. Acc. Chem. Res..

[21] Casillas N., James P., Smyrl W. H. (1995). A novel approach
to combine scanning
electrochemical microscopy and scanning photoelectrochemical microscopy. J. Electrochem. Soc..

[22] Haram S. K., Bard A. J. (2001). Scanning Electrochemical
Microscopy. 42. Studies of
the Kinetics and Photoelectrochemistry of Thin Film CdS/Electrolyte
Interfaces. J. Phys. Chem. B.

[23] Harati M., Jia J., Giffard K., Pellarin K., Hewson C., Love D. A., Lau W. M., Ding Z. (2010). One-pot electrodeposition, characterization
& photoactivity of CIGS thin films for solar cells. Phys. Chem. Chem. Phys..

[24] Esposito D. V., Levin I., Moffat T. P., Talin A. A. (2013). H_2_ evolution
at Si-based metal-insulator-semiconductor photoelectrodes enhanced
by inversion channel charge collection and H spillover. Nat. Mater..

[25] Aaronson B. D. B., Byers J. C., Colburn A. W., McKelvey K., Unwin P. R. (2015). Scanning
Electrochemical Cell Microscopy Platform for Ultrasensitive Photoelectrochemical
Imaging. Anal. Chem..

[26] Conzuelo F., Sliozberg K., Gutkowski R., Grützke S., Nebel M., Schuhmann W. (2017). High-Resolution
Analysis of Photoanodes
for Water Splitting by Means of Scanning Photoelectrochemical Microscopy. Anal. Chem..

[27] Hill, C. M. ; Pan, S. SECM Techniques for Locally Interrogating the Photocatalytic Activity of Semiconducting Materials for Solar-Driven Chemical Transformations. In Scanning Electrochemical Microscopy, 3 ^rd^ ed.; Bard, A. J. , Mirkin, M. V. , Eds.; CRC: Boca Raton, 2022; Ch. 13.

[28] Askarova G., Hesari M., Wang C., Mirkin M. V. (2022). Decoupling Through-Tip
Illumination from Scanning in Nanoscale Photo-SECM. Anal. Chem..

[29] Askarova G., Xiao C., Barman K., Wang X., Zhang L., Osterloh F., Mirkin M. (2023). Photo-scanning Electrochemical
Microscopy
Observation of Overall Water Splitting at a Single Aluminum-Doped
Strontium Titanium Oxide Microcrystal. J. Am.
Chem. Soc..

[30] Askarova G., Hesari M., Barman K., Mirkin M. V. (2023). Visualizing Overall
Water Splitting on Single Microcrystals of Phosphorus-Doped BiVO_4_ by Photo-SECM. ACS Appl. Mater. Interfaces..

[31] Wang Z., Huang Q., Ni C., Bo T., Fan F., Mirkin M. V. (2025). Photoelectrochemical Imaging of Charge
Separation between
MoS_2_ Triangles and Insulating SiO_2_ Support. J. Am. Chem. Soc..

[32] Askarova G., Xiao C., Wu S., Kisslinger K., Osterloh F. E., Mirkin M. V. (2025). Probing Photoelectrochemical Hydrogen
and Oxygen Evolution at Individual Al:SrTiO_3_/Rh_2–y_Cr_y_O_3_ Photocatalyst Particles. Angew. Chem., Int. Ed..

[33] Bae J. H., Nepomnyashchii A. B., Wang X., Potapenko D. V., Mirkin M. V. (2019). Photo-Scanning Electrochemical Microscopy on the Nanoscale
with Through-Tip Illumination. Anal. Chem..

[34] Nie W., Wang X., Wang Z., Gao Y., Chen R., Liu Y., Li C., Fan F. (2021). Identifying
the Role of the Local
Charge Density on the Hydrogen Evolution Reaction of the Photoelectrode. J. Phys. Chem. Lett..

[35] Yu Z., Huang Q., Jiang X., Lv X., Xiao X., Wang M., Shen Y., Wittstock G. (2021). Effect of
a Cocatalyst on a Photoanode in Water Splitting: A Study of Scanning
Electrochemical Microscopy. Anal. Chem..

[36] Chen R., Fan F., Dittrich T., Li C. (2018). Imaging photogenerated charge carriers
on surfaces and interfaces of photocatalysts with surface photovoltage
microscopy. Chem. Soc. Rev..

[37] Lustig D. R., Chen F., Zhang W., Bird O. F., Fajardo J., Ardo S., Hu S., Dukovic G., Talin A. A., Bala Chandran R., Sambur J. B. (2025). Models and Measurements
Quantify Photon Recycling, Charge-Carrier Diffusion and Photon Scattering
Contributions to Photoluminescence in InP Nanowire Arrays. J. Phys. Chem. C.

[38] Nellist M. R., Laskowski F. A. L., Qiu J., Hajibabaei H., Hamann T. W., Boettcher S. W. (2018). Potential-sensing electrochemical
atomic force microscopy for in operando analysis of water-splitting
catalysts and interfaces. Nat. Energy.

[39] Laskowski F. A. L., Oener S. Z., Nellist M. R., Gordon E. M., Boda D. C., Strandwitz N. C., Boettcher S. W. (2020). Nanoscale semiconductor/catalyst
interfaces in photoelectrochemistry. Nat. Mater..

[40] Tachikawa T., Yamashita S., Majima T. (2011). Evidence for Crystal-Face-Dependent
TiO_2_ Photocatalysis from Single-Molecule Imaging and Kinetic
Analysis. J. Am. Chem. Soc..

[41] Roeffaers M. B. J., Sels B. F., Uji-i H., De Schryver F. C., Jacobs P. A., De Vos D. E., Hofkens J. (2006). Spatially
Resolved
Observation of Crystal-Face-Dependent Catalysis by Single Turnover
Counting. Nature.

[42] Pan Z., Yanagi R., Wang Q., Shen X., Zhu Q., Xue Y., Röhr J. A., Hisatomi T., Domen K., Hu S. (2020). Mutually-dependent
kinetics and energetics of photocatalyst/co-catalyst/two-redox
liquid junctions. Energy Environ. Sci..

[43] Sun T., Wang D., Mirkin M. V. (2018). Tunnelling
Mode of Scanning Electrochemical
Microscopy (SECM): Probing Electrochemical Processes at Single Nanoparticles. Angew. Chem., Int. Ed..

[44] Zhao T., Yanagi R., Xu Y., Hu S. (2021). A coating strategy
to achieve effective local charge separation for photocatalytic coevolution. Proc. Natl. Acad. Sci. U.S.A..

[45] Lichterman M. F., Hu S., Richter M. H., Crumlin E. J., Favaro M., Drisdell W., Mayer T., Brunschwig B. S., Lewis N. S., Lewerenz H.-J. (2015). Direct
observation of the energetics at a semiconductor/liquid junction by
operando X-ray photoelectron spectroscopy. Energy
Environ. Sci..

[46] Chen R., Ni C., Zhu J., Fan F., Li C. (2024). Surface photovoltage
microscopy for mapping charge separation on photocatalyst particles. Nat. Protoc..

[47] Takata T., Jiang J., Sakata Y., Nakabayashi M., Shibata N., Nandal V., Seki K., Hisatomi T., Domen K. (2020). Photocatalytic water splitting with a quantum efficiency of almost
unity. Nature.

[48] Pan Z., Röhr J. A., Ye Z., Fishman Z. S., Zhu Q., Shen X., Hu S. (2019). Elucidating
charge separation in
particulate photocatalysts using nearly intrinsic semiconductors with
small asymmetric band bending. Sustainable Energy
Fuels.

[49] Yanagi R., Zhao T., Solanki D., Pan Z., Hu S. (2022). Charge Separation
in Photocatalysts: Mechanisms, Physical Parameters, and Design Principles. ACS Energy Lett..

[50] Kludze A., Bertucci L., Gulati S., Hu S. (2025). Opportunities
for Heterogeneous
Photocatalysis: Quantum Efficiency Enhancement, Selectivity Control,
and Scale Up. Catal. Lett..

[51] Krumov M. R., Simpson B. H., Counihan M. J., Rodríguez-López J. (2018). In Situ Quantification
of Surface Intermediates and Correlation to Discharge Products on
Hematite Photoanodes Using a Combined Scanning Electrochemical Microscopy
Approach. Anal. Chem..

